# Vertical Mandibular Asymmetry in Unilateral Cleft Lip and Palate: A Comparative Analysis With Skeletal Class III and Class I Malocclusions

**DOI:** 10.7759/cureus.76373

**Published:** 2024-12-25

**Authors:** Mahitha Mohan, S. Babukuttan Pillai

**Affiliations:** 1 Department of Orthodontics and Dentofacial Orthopedics, Government Dental College, Thiruvananthapuram, Thiruvananthapuram, IND

**Keywords:** asymmetry index, mandibular asymmetry, orthopantamogram, skeletal class iii, unilateral cleft lip and palate

## Abstract

Introduction

Unilateral cleft lip and palate (UCLP) often leads to maxillary hypoplasia and skeletal Class III malocclusion, with conflicting evidence on mandibular asymmetry. This study evaluated vertical mandibular asymmetry in UCLP patients, comparing them with non-cleft individuals having skeletal Class III and Class I malocclusions.

Methods

Mandibular asymmetry was evaluated using orthopantomograms (OPGs) from 90 subjects divided into three groups of 30 each: UCLP group, non-cleft skeletal Class III, and non-cleft skeletal Class I. Measurements included condylar height, ramal height, condylar + ramal height, gonial angle, antigonial notch depth, mandibular body length, and total mandibular length. These parameters were analyzed to assess vertical mandibular asymmetry. Asymmetry indices were calculated for condylar, ramal, and combined condylar and ramal heights to compare mandibular symmetry across the groups.

Results

The UCLP group showed significant vertical asymmetry, with reduced condylar (-1.267 mm), ramal (-1.700 mm), and combined heights (-2.967 mm) on the cleft side, along with shorter mandibular body (-3.633 mm) and total lengths (-4.067 mm). Asymmetry indices in UCLP were comparable to non-cleft skeletal Class III but significantly differed from non-cleft skeletal Class I for condylar asymmetry (p = 0.042). No significant differences were found between non-cleft skeletal Class III and skeletal Class I groups.

Conclusion

This study revealed significant vertical mandibular asymmetry in UCLP patients, with reduced condylar and ramal heights on the cleft side. UCLP patients exhibited greater condylar asymmetry compared to skeletal Class I individuals, but no significant differences were found with non-cleft skeletal Class III patients. Early intervention is crucial to address these growth disturbances and improve patient outcomes.

## Introduction

Orofacial clefts are the most prevalent craniofacial birth defects in humans. The global incidence of cleft lip and palate (CLP) anomalies is estimated to be between 0.8 and 1.6 per 1,000 live births. In India, the incidence ranges from approximately 0.93 to 1.3 per 1,000 live births, with orofacial clefts being more prevalent in males (53.83%) than in females (46.17%) [1]. People with CLP typically show distinct facial growth compared to individuals without these conditions [2]. They are often predisposed to developing skeletal Class III malocclusion, featuring an underdeveloped maxilla along with anterior and posterior crossbites. This is typically due to surgical scar and/or congenital growth deficiency [3].

Mandibular growth in those with unilateral cleft lip and palate (UCLP) is generally not influenced by the cleft alone. However, when the mandible aligns with an asymmetrical maxilla, it may result in a unilateral posterior crossbite, which is often associated with changes in muscle function [4]. These altered muscle functions can affect mandibular growth, possibly resulting in structural changes. Many studies have reported asymmetrical growth of the maxilla in individuals with UCLP [5]. Some research [6] indicates the presence of mandibular asymmetry in such patients, whereas other studies [7,8] have found no significant evidence of mandibular asymmetry in this group.

Habets et al. introduced a technique for assessing vertical condylar and ramal asymmetry by utilizing specific linear and angular measurements on orthopantomogram (OPG) tracings. [9,10]. This approach has been widely adopted by researchers to examine condylar asymmetry in various conditions, including unilateral and bilateral posterior crossbites [11,12], temporomandibular disorders, early removal of bilateral first molars [13], and in individuals with Class II and Class III [14] malocclusions. A study evaluating three-dimensional facial morphology in children with unilateral and bilateral CLP, as well as isolated cleft palate, highlighted varying degrees and distinct patterns of asymmetry characteristic of different CLP deformities [2].

In many earlier studies, control groups typically included non-cleft individuals with normal occlusion. However, it is well-established that individuals with CLP often exhibit varying degrees of skeletal Class III malocclusion, typically accompanied by maxillary hypoplasia [15]. Consequently, CLP patients tend to present more severe sagittal discrepancies and transverse asymmetries than non-cleft individuals with normal occlusion. Given that skeletal Class III malocclusion is commonly seen in CLP patients, it is essential to compare these individuals with those having skeletal Class III malocclusion to better understand the unique characteristics of mandibular asymmetry in CLP. Thus, the aim of this study was to assess vertical mandibular asymmetry in patients with UCLP and compare the findings with those of non-cleft individuals who had skeletal Class III and Class I malocclusions.

## Materials and methods

Study design and setting

This comparative study was conducted using clinical and radiographic records obtained as part of routine orthodontic diagnosis at a tertiary dental healthcare center in India. Institutional Ethics Committee approval was obtained (IEC approval no. DCT/IEC/SS/24/11, dated 10/01/2024). Data were collected retrospectively from patient records in the Department of Orthodontics.

Sample size calculation

The sample size for this study was determined through a power analysis to ensure statistical reliability. A power level of 80% (Z_β_ = 0.84) and a 5% type I error rate (Z_α/2_ = 1.96) were applied. The mean difference between groups, based on existing literature, was calculated to be 4.18 [16,17]. The pooled standard deviation (SD) was 5.79. These factors indicated that a minimum of 30 subjects per group was required for the study.

Inclusion and exclusion criteria

This study analyzed diagnostic records of 90 subjects aged 11 to 25 years, divided into three groups of 30 individuals each, with an equal distribution of males and females in each group. Group I consisted of subjects with complete unilateral cleft lip, alveolus, and palate accompanied by maxillary hypoplasia and a Class III skeletal relationship. To qualify for this group, participants had to meet specific criteria, including no systemic diseases or craniofacial/neuromuscular deformities and no prior orthodontic treatment. Group II comprised non-cleft individuals with skeletal Class III malocclusion, adhering to similar inclusion criteria as Group I, excluding the presence of a cleft lip or palate.

Group III consisted of non-cleft individuals with skeletal Class I malocclusion, characterized by normal growth and development, a bilateral Angle's Class I molar relationship, moderate crowding or spacing, and good facial symmetry. Additionally, these participants had no significant medical history and no previous orthodontic, prosthodontic, maxillofacial, or plastic surgery treatments.

To ensure homogeneity among the study groups regarding craniofacial development and systemic health, exclusion criteria applied to all groups included syndromic CLP, underlying systemic diseases, and any history of orthodontic treatment.

Methodology

Mandibular asymmetry in non-cleft skeletal Class III subjects and subjects with UCLP was evaluated, and compared with that of non-cleft skeletal Class I subjects using OPGs. The outline of the entire mandible was traced on acetate paper. Condylar height (CH), ramal height, condylar + ramal height, gonial angle, depth of the antigonial notch, mandibular body length, and total mandibular length were measured on the OPG tracings [10].

**Figure 1:**
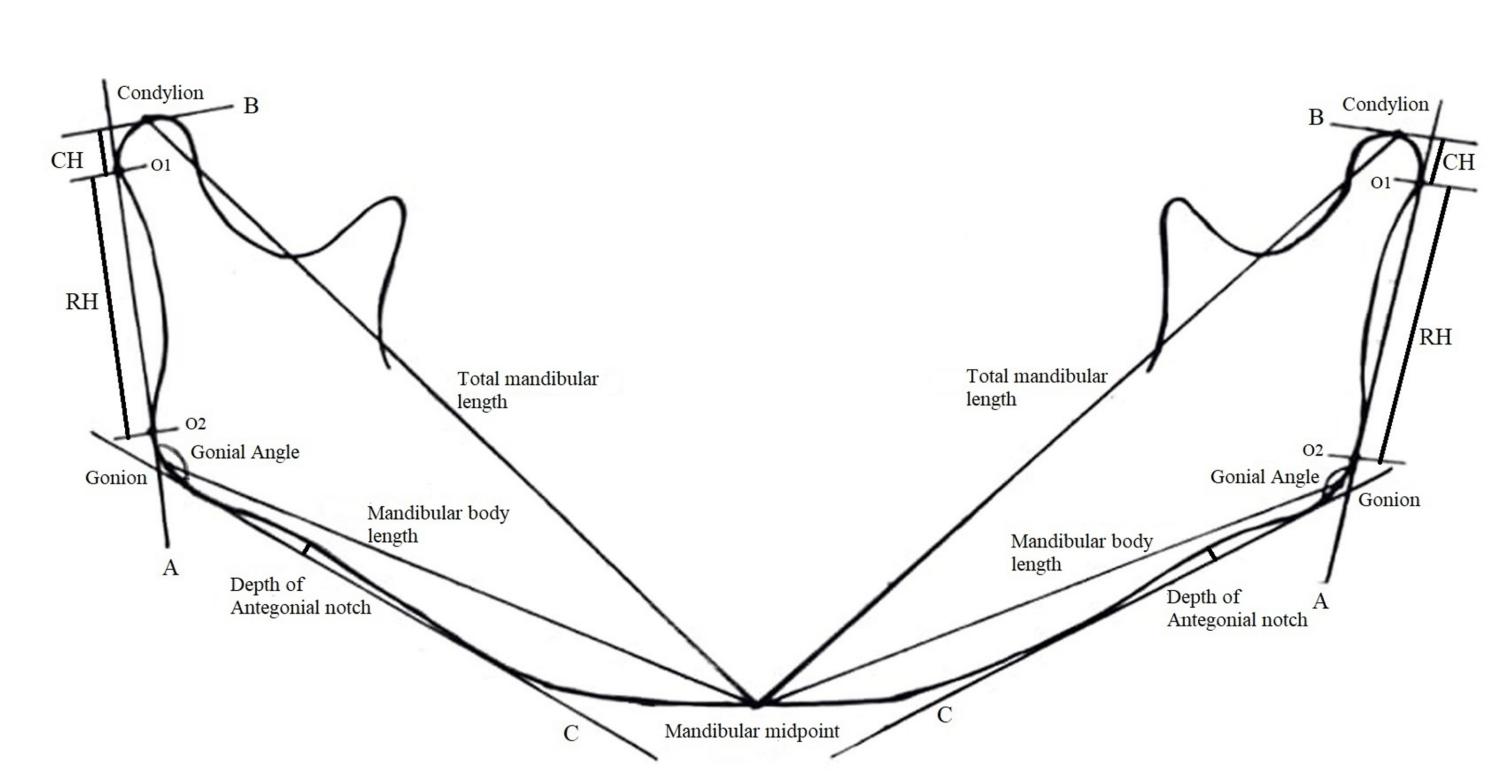
Orthopantomogram (OPG) tracing showing the measurements CH - condylar height; RH -ramal height; O1 - the most lateral point of the condylar image; O2 - the most lateral point of the ascending ramus image; A-line - ramus tangent; B-line - perpendicular to the most superior point of the condylar image; C-line - a tangent along the mandibular corpus. Original tracing was created during the study.

On the OPG tracing, a line (A-line, the ramus tangent) was drawn between the most lateral points of the condylar image (O1) and the ascending ramus image (O2). From the most superior point of the condylar image, a perpendicular line (B-line) was drawn to the A-line. The vertical distance from this perpendicular line to O1, as projected on the ramus tangent, was measured and termed the CH. The distance between O1 and O2 was referred to as the ramus height (RH) (Figure 1).

A C-line was constructed as a tangent along the mandibular corpus on each side, and the angle between the A-line and C-line was measured as the gonial angle. The maximal depth from the C-line to the lower border of the mandible was recorded as the depth of the antegonial notch. Mandibular body length was measured as the distance from the gonion to the mandibular mid-point, while total mandibular length was measured from the condylion to the mandibular mid-point (Figure 1).

For the estimation of condylar, ramal, and condylar + ramal asymmetry, the following indices were used [10].

The condylar asymmetry index was determined by dividing the difference between the right and left CHs by their total sum and multiplying the result by 100. Similarly, the ramal asymmetry index was calculated by dividing the difference between the right and left ramal heights by their total sum and multiplying by 100. The combined condylar and ramal asymmetry index was obtained by dividing the difference between the total of the right condylar and ramal heights and the total of the left condylar and ramal heights by the overall sum of the condylar and ramal heights on both sides, then multiplying the result by 100.

Statistical analysis

The data collected were entered in Microsoft Excel (Microsoft® Corp., Redmond, WA). Statistical analysis was done using SPSS software (version 25.0; IBM Corp., Armonk, NY). Quantitative variables were expressed in terms of mean and SD. Intragroup comparison within the UCLP group was done using the Wilcoxon sign rank test. A comparison of asymmetry indices among the three groups was done using the Kruskal-Wallis test. Multiple comparisons of parameters among the groups were done using the Kruskal-Wallis test and Bonferroni post hoc test. The level of significance was set at p < 0.05.

## Results

In this study, vertical mandibular asymmetry was evaluated by comparing measurements across three groups: patients with UCLP, non-skeletal Class III malocclusions, and non-skeletal Class I malocclusions. The analyzed parameters included CH, ramal height, combined condylar and ramal height, gonial angle, antegonial notch depth, mandibular body length, and total mandibular length, with mean values presented in Table 1.

**Table 1:** Mean values of measured parameters UCLP - unilateral cleft lip and palate

Parameters	UCLP		Non-skeletal Class III		Non-skeletal Class I	
	Cleft side	Normal side	Right side	Left side	Right side	Left side
Condylar height	5.93	7.2	6.93	6.9	6.77	6.77
Ramal height	45.77	47.47	46.03	45.83	45.57	45.57
Condylar + ramal height	51.70	54.67	52.97	52.73	52.33	52.33
Gonial angle	131.33	128.50	130.73	130.53	127.80	127.80
Antegonial notch	1.87	1.17	1.87	1.83	1.83	1.83
Mandibular body length	89.13	92.77	90.67	90.60	85.70	85.70
Total mandibular length	124.23	128.3	130.40	130.40	129.93	129.93

The intragroup comparison within the UCLP group using the Wilcoxon signed-rank test revealed significant vertical asymmetry between the cleft and non-cleft sides for all parameters. Specifically, the cleft side exhibited reduced CH (-1.267 mm), ramal height (-1.700 mm), and combined condylar and ramal height (-2.967 mm), alongside an increased gonial angle (2.833°) and greater antegonial notch depth (0.700 mm). Additionally, both mandibular body length and total mandibular length were significantly shorter on the cleft side, by -3.633 mm and -4.067 mm, respectively (Table 2).

**Table 2:** Intragroup comparison within the UCLP group using the Wilcoxon signed-rank test *p-value < 0.05 - statistically significant CI - confidence interval; SD - standard deviation; SE - standard error; UCLP - unilateral cleft lip and palate

Groups	Mean	SD	SE	95% CI		Test statistics	p-value
				Lower	Upper		
Condylar height - cleft side and normal side	-1.267	0.907	0.166	-1.605	-0.928	300.00	0.000*
Ramal height - cleft side and normal side	-1.700	1.343	.245	-2.201	-1.199	399.00	0.000*
Condylar height + ramal height - cleft side and normal side	-2.967	1.426	.260	-3.499	-2.434	435.00	0.000*
Gonial angle - cleft side and normal side	2.833	2.854	.521	1.768	3.899	41.00	0.000*
Antegonial notch - cleft side and normal side	0.700	0.702	0.128	0.438	0.962	22.00	0.000*
Mandibular body length - cleft side and normal side	-3.633	2.341	.427	-4.508	-2.759	406.00	0.00*
Total mandibular length - cleft side and normal side	-4.067	2.970	.542	-5.176	-2.958	431.5	0.000*

The comparison of asymmetry indices among the groups using the Kruskal-Wallis test showed that the UCLP group had mean indices of 0.257 for condylar asymmetry, 0.222 for ramal asymmetry, and 0.220 for combined asymmetry, indicating that while some asymmetry exists, it is not significantly different from non-cleft Class III or Class I malocclusions (Table 3).

**Table 3:** Comparison of asymmetry indices among the three groups using the Kruskal-Wallis test

Asymmetry indices	Unilateral	Non-skeletal Class III	Non-skeletal Class I	Test statistics	p-value
Condylar asymmetry	0.257	0.00	0.00	3.610	0.165
Ramal asymmetry	0.222	0.00	0.00	1.826	0.401
Condylar + ramal asymmetry	0.220	0.00	0.00	4.441	0.109

However, the Kruskal-Wallis and Bonferroni post hoc tests identified a significant difference in the condylar asymmetry index between the UCLP and non-cleft Class I groups (mean difference 4.124; p = 0.042), while no significant differences were found between the UCLP and non-cleft Class III groups or between non-cleft Class III and Class I groups. Thus, significant asymmetry differences were primarily noted between UCLP and non-cleft Class I (Table 4).

**Table 4:** Multiple comparisons using Bonferroni post hoc test *p-value < 0.05 - statistically significant CI - confidence interval; SE - standard error

Dependent variable	Groups	Groups	Mean difference between groups	SE	Test statistics	p-value	95% CI	
							Lower bound	Upper bound
Condylar asymmetry index	UCLP	Non-skeletal Class III	3.867	1.641	3.610	0.062	-0.1400	7.8740
	Non-skeletal Class III	Non-skeletal Class I	4.124	1.641		0.042*	0.1167	8.1306
		Non-skeletal Class I	0.257	1.641		1.000	-3.7503	4.2636
Ramal asymmetry index	UCLP	Non-skeletal Class III	0.043	0.353	1.826	1.000	-0.8194	0.9054
	Non-skeletal Class III	Non-skeletal Class I	0.265	0.353		1.000	-0.5970	1.1277
		Non-skeletal Class I	0.222	0.353		1.000	-0.6400	1.0847
Condylar + ramal asymmetry index	UCLP	Non-skeletal Class III	0.737	0.458	4.441	0.333	-0.3800	1.8540
	Non-skeletal Class III	Non-skeletal Class I	0.957	0.458		0.118	-0.1604	2.0737
		Non-skeletal Class I	0.220	0.458		1.000	-0.8974	1.3367

## Discussion

Vertical mandibular asymmetries can lead to functional issues, including compromised occlusion and temporomandibular joint (TMJ) disorders, as well as aesthetic concerns [7]. Understanding the extent and nature of vertical mandibular asymmetry in UCLP patients is essential for effective treatment planning and intervention. In this study, vertical mandibular asymmetry in the UCLP group was compared to that of skeletal Class III patients, as both groups display maxillary deficiencies despite differing etiologies. The skeletal Class I group serves as a control, providing a baseline to assess the degree of asymmetry in both UCLP and Class III patients. Cases with associated medical conditions or syndromes were excluded from the study, as conditions like Treacher Collins syndrome, Goldenhar syndrome, Pierre Robin sequence, and Stickler syndrome can cause notable facial asymmetry due to craniofacial deformities, altered growth patterns, and secondary features such as mandibular hypoplasia or midface retrusion. These factors could influence mandibular growth and asymmetry, potentially affecting the study's outcomes.

The results of this study showed significant vertical mandibular asymmetries in the UCLP group, especially on the cleft side, where the vertical dimensions of the mandible were often reduced. The observed reduction in condylar and ramal height on the cleft side compared to the non-cleft side is consistent with that of previous studies [16,18]. These differences are indicative of growth disturbances that begin early in life as a result of the cleft deformity and its associated surgical treatment. The cleft condition can negatively impact muscle function, resulting in altered biomechanics within the TMJ, which ultimately affects the vertical growth of both the condylar and ramal regions [7].

The significant increase in the gonial angle on the cleft side reported in this study may indicate compensatory adaptations in the mandible, a finding that aligns with previous studies [16,19]. Additionally, the reduction in both mandibular body length and total mandibular length on the cleft side further supports the notion that the cleft condition disrupts normal mandibular development. Another finding in the study was the increased depth of the antegonial notch on the cleft side, which may indicate underdevelopment of the mandible in that area, possibly due to impaired vertical growth as it compensates for the differences between the cleft and non-cleft sides. When comparing the asymmetry indices among the UCLP, non-cleft skeletal Class III, and non-cleft skeletal Class I groups, significant differences were mainly noted between the UCLP and non-cleft Class I malocclusion groups, especially in condylar asymmetry. This may be attributed to compensatory adjustments for pre-existing cranial base and naso-maxillary complex asymmetries in UCLP, as well as muscular tension from postoperative scarring [18].

Kurt et al. [19] applied the method of Habets et al. to assess the mandibular asymmetry in CLP patients and found no statistically significant differences among unilateral (UCLP) and bilateral cleft lip and palate (BCLP) groups and a control group with normal occlusion. However, a statistically significant increase in gonial angle was observed on the cleft side in the UCLP group. In a separate study, Jena et al. [16] examined the effects of sagittal maxillary growth hypoplasia severity on mandibular asymmetry in UCLP patients. They concluded that the mandible was significantly asymmetrical in those with near-normal sagittal maxillary growth, whereas it was nearly symmetrical in subjects with severe maxillary growth hypoplasia.

OPG was used in this study because it provides a bilateral view that effectively evaluates the vertical asymmetry of the condyle and ramus. Additionally, it is cost-effective, has low radiation exposure, and is a routine diagnostic tool. The method by Habets et al. was selected for its proven reliability in using OPGs to assess vertical asymmetry, ensuring consistency and accuracy in comparing the study groups [9].

This study highlights the significance of early diagnosis and intervention in addressing vertical mandibular asymmetry in patients with UCLP, as untreated asymmetries may result in functional issues like TMJ dysfunction and aesthetic challenges. The results also emphasize the necessity for customized treatment strategies for cleft patients, taking into account their distinct growth disturbances in comparison to non-cleft individuals with similar malocclusions.

Limitations and future directions

One limitation of this study is its relatively small sample size, which may have limited the capacity to identify significant differences in certain measures, especially regarding ramal asymmetry. Furthermore, the cross-sectional design does not capture changes in mandibular asymmetry over time. Future research should involve larger sample sizes and incorporate longitudinal analyses to gain a deeper understanding of how mandibular asymmetry develops in cleft patients. Additionally, studies should focus on UCLP patients with medical conditions and examine their influence on variations in facial growth patterns.

## Conclusions

This study demonstrates significant vertical mandibular asymmetry in UCLP patients, characterized by reductions in condylar and ramal heights, as well as mandibular body length on the cleft side. These findings underscore the distinct vertical growth disturbances in UCLP compared to non-cleft individuals. Comparisons revealed that UCLP patients have notably greater condylar asymmetry than those with skeletal Class I malocclusions, while no significant differences were observed between UCLP and non-cleft skeletal Class III patients. The results highlight the necessity of early intervention for vertical asymmetry in UCLP patients to prevent functional and aesthetic complications. Clinicians should take these unique growth disturbances into account when planning treatment, focusing on correcting condylar and ramal height discrepancies to enhance patient outcomes.
